# In vitro effects of 5-Hydroxy-L-tryptophan supplementation on primary bovine mammary epithelial cell gene expression under thermoneutral or heat shock conditions

**DOI:** 10.1038/s41598-022-07682-7

**Published:** 2022-03-09

**Authors:** Sena L. Field, Véronique Ouellet, Celeste M. Sheftel, Laura L. Hernandez, Jimena Laporta

**Affiliations:** 1 Department of Animal and Dairy Sciences, University of Wisconsin-Madison, Madison, WI 53706 USA; 2 Department of Animal Sciences, Université Laval, Québec City, QC Canada

**Keywords:** Cell biology, Molecular biology

## Abstract

Serotonin (5-HT) is an autocrine-paracrine molecule within the mammary gland regulating homeostasis during lactation and triggering involution after milk stasis. Exposure of dairy cows to hyperthermia during the dry period alters mammary gland involution processes leading to reduced subsequent yields. Herein, primary bovine mammary epithelial cells (pBMEC) under thermoneutral (TN, 37 °C) or heat shock (HS, 41.5 °C) conditions were cultured with either 0, 50, 200, or 500 μM 5-Hydroxy-L-tryptophan (5-HTP; 5-HT precursor) for 8-, 12- or 24-h. Expression of 95 genes involved in 5-HT signaling, involution and tight junction regulation were evaluated using a Multiplex RT-qPCR BioMark Dynamic Array Circuit. Different sets of genes were impacted by 5-HTP or temperature, or by their interaction. All 5-HT signaling genes were downregulated after 8-h of HS and then upregulated after 12-h, relative to TN. After 24-h, apoptosis related gene, *FASLG,* was upregulated by all doses except TN-200 μM 5-HTP, and cell survival gene, *FOXO3,* was upregulated by HS-50, 200 and 500 μM 5-HTP, suggesting 5-HTP involvement in cell turnover under HS. Supplementing 5-HTP at various concentrations in vitro to pBMEC modulates the expression of genes that might aid in promoting epithelial cell turn-over during involution in dairy cattle under hyperthermia.

## Introduction

Dairy cows are subjected to frequent milking intervals during the lactation period, typically followed by an abrupt cessation of milking to initiate a 6 to 8-week non-lactating phase between two subsequent lactations, referred to as the “dry period”. During the dry period, senescent mammary epithelial cells (MEC) are replaced with newly synthesized MECs which enables the udder to maximize milk synthesis in the subsequent lactation^1^. Even though the dry period is not essential for milk production, continuous milking or significantly reducing the dry period length diminishes the next lactation yield up to 20%^2–4^.

Mammary gland involution is a complex, multi-step, highly regulated process triggered by milk-stasis-induced accumulation of numerous local factors within the milk. The autocrine actions of local factors along with reduced systemic endocrine signals, result in a decline in milk synthesis^5^. Bioactive factors, such as parathyroid hormone- related protein, transforming growth factor, insulin-like growth factor and serotonin (5-hydroxytryptamine, 5-HT), accumulate in the milk to elicit microstructure modifications initiating a remodeling of extracellular matrix and cytoskeleton components, as well as a disruption of MEC tight junctions and downregulating milk protein gene expression^6–11^. As the dry period advances and parturition approaches, mammary growth is driven by autocrine signals and systemic mammogenic hormones, such as prolactin, which activates signal transducer and activator of transcription (STAT) factors. During the latter phase of the dry period, STAT5 increases MEC proliferation and differentiation in preparation for milk synthesis^12^. The efficient removal of senescent MEC to allow the synthesis of new MEC in this redevelopment phase is crucial to maximize milk yield in the next lactation^13^. Environmental factors, such as heat stress, have been shown to alter this highly regulated cellular process, leading to a less productive mammary gland^13,14^. Specifically, exposure of dairy cows to dry period heat stress extends cell death signals beyond involution compromising mammary gland growth prior to parturition and ultimately decreasing milk yield in the next lactation^15,16^.

5-HT is a biogenic monoamine derived from L-tryptophan and converted by tryptophan hydroxylase (TPH1, rate limiting enzyme) to 5-Hydroxy-L-tryptophan (5-HTP, serotonin precursor), which is then converted to 5-HT by the aromatic amino acid decarboxylase (AADC, a ubiquitous enzyme). 5-HT can be taken into the cell via the 5-HT reuptake transporter (SERT) and metabolized to 5-Hydroxyindoleacetic acid (5-HIAA). The human, rodent, and bovine mammary glands express a dynamic and unique array of 5-HT receptors, as well as SERT and TPH1^9,17,18^. The participation of MEC serotonergic system is well established in lactation and involution, where it acts as a homeostatic regulator of lactation and a potent feedback inhibitor of lactation, respectively^19,20^. In human epithelial cells cultured in vitro*,* 5-HT accelerates involution by acting through the 5-HT7 receptor to disrupt tight junction permeability on MEC decreasing transepithelial electrical resistance^9,21^. Hernandez et al.^20^ demonstrated serotonin’s role as a negative regulator of lactation by downregulating the expression of milk protein genes in cultured primary bovine mammary epithelial cells (pBMEC). Additionally, intramammary infusions of a selective serotonin reuptake inhibitor, fluoxetine, reduced milk synthesis through an increase in tight junction permeability and increase in plasma lactose concentrations^10^.

The objectives of this study were to determine the effects of 5-HTP treatment, the precursor for 5-HT synthesis, to pBMEC cultured under thermoneutral or heat shock conditions on the expression of tight junction, extracellular matrix remodeling, apoptosis, autophagy, and cell proliferation genes, and to evaluate pBMEC ductal branching morphogenesis under these conditions. We hypothesized incremental doses of 5-HTP would elicit a dose-dependent response in pBMEC gene expression, and that increasing incubation temperature and time exposure would impact pBMEC gene expression responses and cell morphology. Understanding the underpinnings of the 5-HT system within the mammary tissue under hyperthermic conditions might offer a potential avenue for the regulation of mammary function to achieve optimal milk synthesis in warm climates.

## Results

### Effect of temperature: 8-h incubation

**Figure 1:**
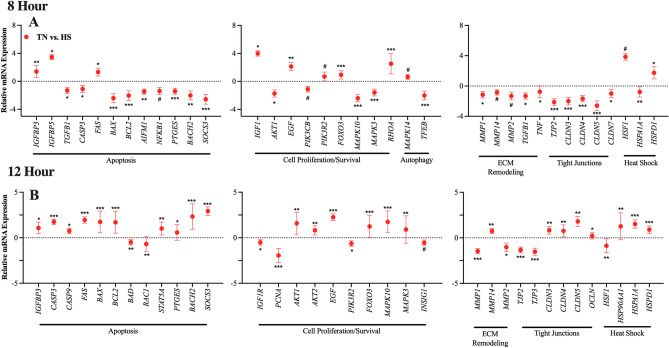
Main effect of temperature. Gene expression of primary bovine mammary epithelial cells (pBMEC) after 8- or 12-h incubation at thermoneutral (37 °C, TN) or heat shock (41.5 °C, HS) culture conditions. Data is presented as relative mRNA expression (∆∆Ct = ∆Ct TN (CON) − ∆Ct HS)) ± standard error of the mean (SEM). Differentially expressed genes are grouped by biological function. After 24-h incubation at HS conditions, 9 genes (*PTGES*, *5-HT2C*, *ATG3*, *AKT1*, *PIK3R2*, *PRKACA*, TNF, *CLDN1*, *OCLN*) were differentially expressed, relative to TN (graph panel not shown). Positive and negative ∆∆Ct indicates gene upregulation and downregulation, respectively. Significance declared at (*) *P* value ≤ 0.05, (**) *P* value ≤ 0.001 (***) *P* value ≤ 0.0001 and (#) denotes a statistical tendency at 0.05 < *P* value ≤ 0.10.

Cells were harvested after 8-h incubation under TN or HS conditions. Refer to Supplemental Table 2 for a full list of all significant genes for the main effect of temperature. Gene expression was analyzed and differentially expressed genes are reported as ∆∆Ct of HS relative to TN (Fig. 1A). An 8-h incubation under HS conditions downregulated eight genes related to apoptosis (*TGFB1, CASP3, BAX, BCL2, AIFM1, PTGES, BACH2,* and *SOCS; P* < 0.05), and *NFKB1* tended to be downregulated (*P* = 0.06). Three genes related to apoptosis (i.e., *IGFBP3, IGFBP5* and *FAS; P* < 0.04) were upregulated, relative to TN. Autophagy related genes (i.e., *RHOA* and *MAPK14; P* < 0.1) were upregulated and *TFEB* (*P* = 0.0002) downregulated, relative to TN. Three genes related to cell proliferation and survival (i.e., *AKT1, MAPK10* and *MAPK3*; *P* < 0.02) were downregulated and *PIK3CB* tended to be downregulated (*P* = 0.1), compared to TN. Three genes related to cell proliferation/survival (i.e., *IGF1, EDF* and *FOXO3; P* < 0.01) were upregulated and *PIK3R2* (*P* = 0.10) tended to be upregulated, compared to TN. Genes related to ECM remodeling were all downregulated (*MMP1, TNF* and *TGFB1; P* < 0.05), as well as *MMP14* and *MMP2* (*P* < 0.08) tended to be downregulated, compared to TN. Genes related to tight junctions, *TJP2, CLDN3, CLDN4, CLDN5* and *CLDN7* (*P* < 0.02) were all downregulated, compared to TN. Genes related to heat shock were upregulated (i.e., *HSPD1* and *HSF1*; *P* < 0.07) and *HSPA1A* (*P* = 0.008) was downregulated, compared to TN.

**Figure 2:**
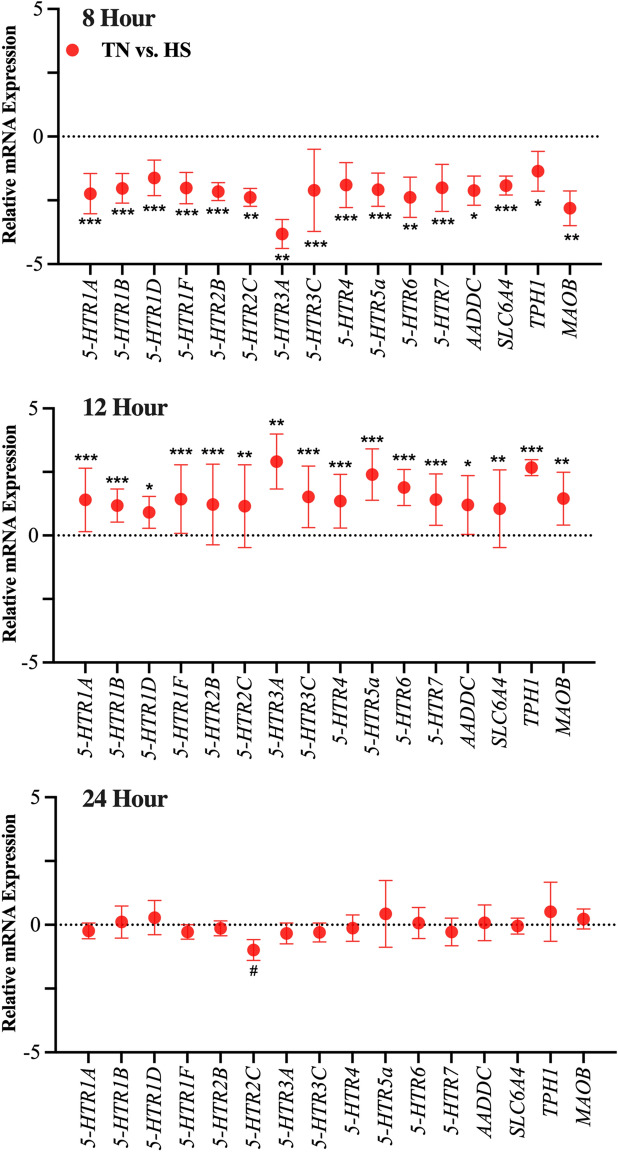
Main effect of incubation temperature. Gene expression of primary bovine mammary epithelial cells (pBMEC) exposure to either thermoneutral (37 °C, TN) or heat shock (41.5 °C, HS) culture conditions for 8-, 12- or 24-h. Data is presented as relative mRNA expression (∆∆Ct = ∆Ct TN (CON) − ∆Ct (HS)) ± standard error of the mean (SEM). Positive and negative ∆∆Ct indicates upregulation and downregulation, respectively. Significance declared at (*) *P* value ≤ 0.05, (**) *P* value ≤ 0.001 (***) *P* value ≤ 0.0001 and (#) denotes a statistical tendency at 0.05 < *P* value ≤ 0.10.

Noticeably, after an 8-h incubation under HS, all serotonin receptors (i.e., *5-HTR1A, 5-HTR1B, 5-HTR1D, 5-HTR1F, 5-HTR2B, 5-HTR2C, 5-HTR3A, 5-HTR3C, 5-HTR4, 5-HTR5a, 5-HTR6* and *5-HTR7*; *P* < 0.005, Fig. 2) were downregulated, compared to TN. Similarly, the genes for 5-HT transporter, serotonin rate-limiting enzyme, the ubiquitous enzyme that converts 5-HTP to serotonin, and one of serotonin degrading enzymes (*SLC6A4, TPH1, AADDC,* and *MAOB;* respectively) were all downregulated (*P* < 0.02, Fig. 2) compared to TN.

### Effect of temperature: 12-h incubation

Cells were harvested after a 12-h incubation under TN or HS conditions. Gene expression was analyzed and differentially expressed genes are reported as the ∆∆Ct with HS relative to TN (Fig. 1B). Ten genes related to apoptosis were upregulated (i.e., *IGFBP3, CASP3, CASP9, FAS, BAX, BCL2, PTGES, BACH2, SOCS3* and *STAT5A; P* < 0.05), while *BAD* and *RAC1* (P < 0.003) were downregulated, compared to TN. Six genes related to cell proliferation/survival (i.e., *AKT1, AKT2, EGF, FOXO3, MAPK10* and *MAPK3*; *P* < 0.008) were upregulated, while *PCNA, PIK3R2* (*P* < 0.01*)* were downregulated and *IGF1R* and *INSIG1* (*P* < 0.08) tended to be downregulated. ECM remodeling genes, *MMP1* and *MMP2* (P < 0.03) were downregulated, while MMP14 (*P* = 0.006) was upregulated, compared to TN. Tight Junction genes, *TJP2* and *TJP3* (*P* < 0.004), were downregulated and *CLDN3, CLDN4, CLD5* and *OCLN* (*P* < 0.04) were upregulated, compared to TN. Three genes related to heat shock (i.e., *HSP90AA1, HSPA1A* and *HSPD1*; *P* < 0.004) were upregulated, while *HSF1* (*P* = 0.007) was downregulated, compared to TN.

After a 12-h incubation, all serotonin receptors (i.e., *5-HTR1A, 5-HTR1B, 5-HTR1D, 5-HTR1F, 5-HTR2B, 5-HTR2C, 5-HTR3A, 5-HRT3C, 5-HTR4, 5-HTR5a, 5-HTR6* and *5-HTR7*; *P* < 0.01, Fig. 2) were upregulated, compared to TN. Similarly, *SLC6A4*, *TPH1*, *AADDC* and *MOAB* were all upregulated (*P* < 0.04, Fig. 2), compared to TN.

### Effect of temperature: 24-h incubation

Cells were harvested after a 24-h incubation at TN or HS conditions. Gene expression was analyzed and differentially expressed genes are reported as the ∆∆Ct (HS relative to TN conditions, Supplemental Table 2). Compared to the 8- and 12-h time points, the number of significant differentially expressed genes was greatly reduced after 24 h. An apoptotic related gene, *PTGES*, was downregulated (*P* = 0. 01) and a 5-HT receptor, *5-HT2C*, tended to be downregulated (*P* = 0.08, Fig. 2). An autophagy related gene, *ATG3*, tended to be downregulated (*P* = 0.08). One gene related to cell proliferation/survival, *AKT1* (*P* = 0.06)*,* tended to be downregulated, while *PIK3R2* and *PRKACA* (*P* < 0.05), were upregulated. An ECM remodeling related gene, *TNF*, was downregulated (*P* = 0.03), and two tight junction related genes, *CLDN1* and *OCLN* (*P* < 0.05), were downregulated in HT compared relative to TN.

### Effect of 5-HTP dose: 8-h incubation

Cells were harvested after an 8-h incubation with various 5-HTP doses. Gene expression was analyzed and differentially expressed genes are reported as the ∆∆Ct (5-HTP doses 50, 200 and 500 μM relative to 5-HTP dose 0 μM; data not shown). Only two genes were impacted at 8-h for the main effect of 5-HTP. All doses downregulated *BCL2*, an apoptotic related gene (*P* < 0.03). The 5-HTP doses, 50 and 200 μM, downregulated the expression of *MAPK10*, a cell proliferation/survival related gene (*P* < 0.02).

### Effect of 5-HTP dose: 12-h incubation

**Figure 3:**
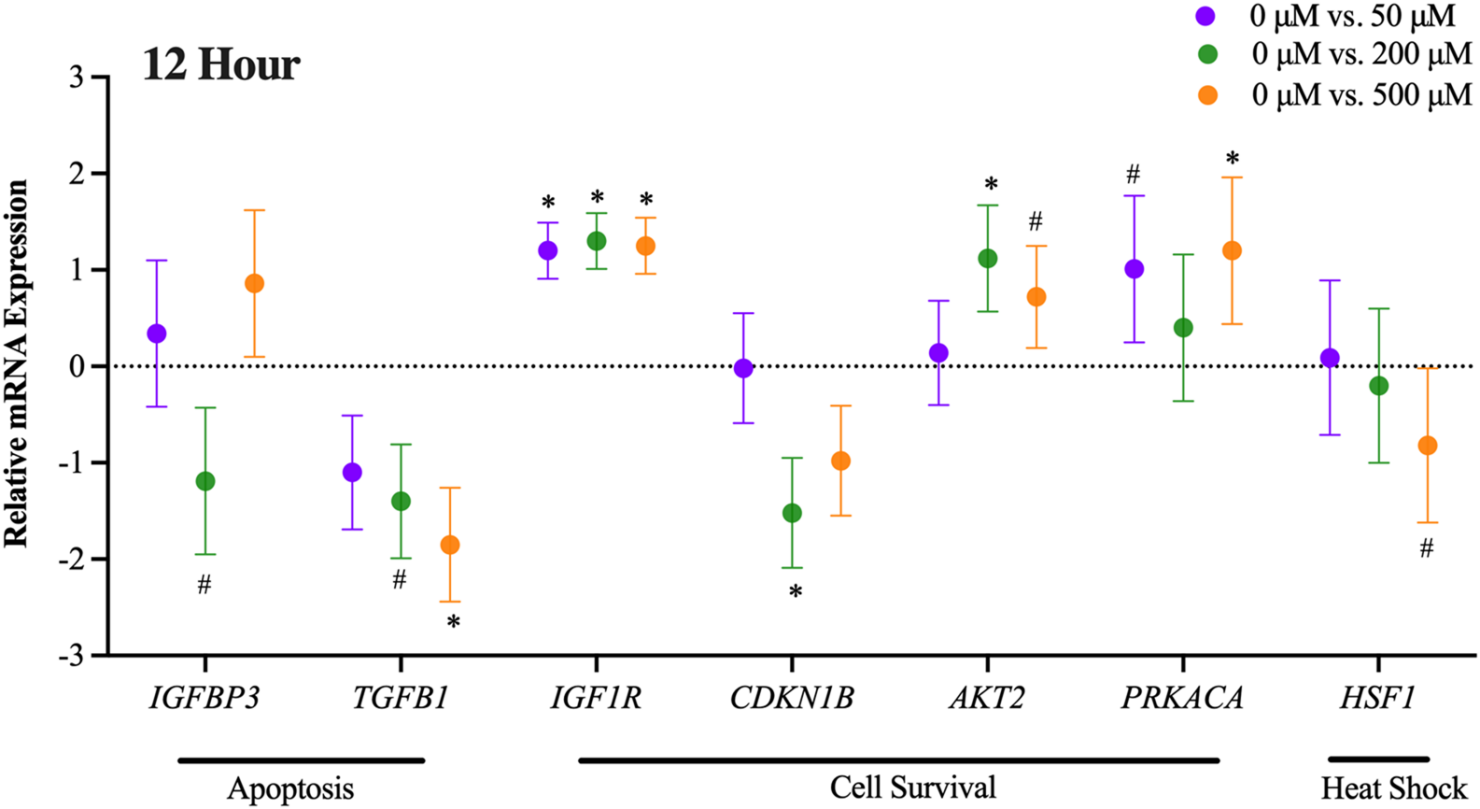
Main effect of 5-Hydroxy-L-tryptophan (5-HTP) dose. Gene expression of primary bovine mammary epithelial cells (pBMEC) cultured with 50, 200 or 500 μM of 5-HTP for 12-h. Data is presented as relative mRNA expression (∆∆Ct = ∆Ct Dose 0 (CON) − ∆Ct Dose 50, 200 or 500)) ± standard error of the mean (SEM). Positive and negative ∆∆Ct indicates upregulation and downregulation, respectively. Significance declared at *P* value ≤ 0.05 (*) and (#) denotes a statistical tendency at 0.05 < *P* value ≤ 0.10.

Cells were harvested after a 12-h incubation with various 5-HTP doses. Gene expression was analyzed and differentially expressed genes are reported as the ∆∆Ct (5-HTP doses 50, 200 and 500 μM relative to 5-HTP 0 μM dose; Fig. 3). Refer to Supplemental Table 3 for a full list of all significant genes for the main effect of 5-HTP dose at 12-h. The 200 μM dose tended to downregulate *IGFBP3* (*P* = 0.1). and *TGFB1*(*P* = 0.08), while the 500 μM dose downregulated *TGFB1* (*P* = 0.02), expression, relative to 0 μM dose. Cell proliferation gene, *IGF1R*, was upregulated by all 5-HTP doses (*P* = 0.005) and *CDKN1B* was downregulated only by the 200 μM dose (*P* = 0.05). The 200 μM dose upregulated and the 500 μM dose tended to upregulate the expression of *AKT2* (*P* < 0.08). Cell proliferation/survival gene, *PRKACA*, tended to be upregulated by the 50 μM dose and was upregulated by the 500 μM dose (*P* < 0.07). Heat shock related gene, *HSF1*, tended to be downregulated by the 500 μM dose (*P* = 0.06).

### Effect of 5-HTP dose: 24-h incubation

**Figure 4:**
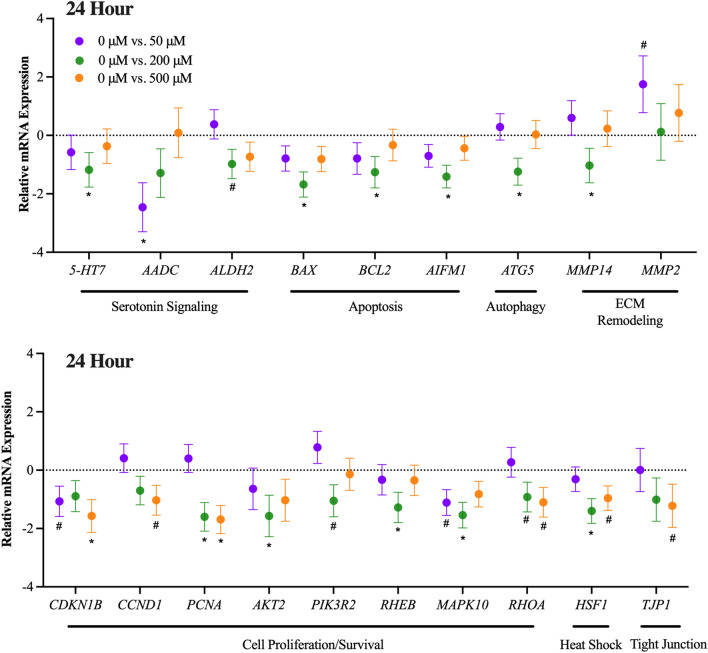
Main effect of 5-Hydroxy-L-tryptophan (5-HTP) dose. Gene expression of primary bovine mammary epithelial cells (pBMEC) cultured with 50, 200 or 500 μM of 5-HTP for 24-h. Data is presented as relative mRNA expression (∆∆Ct = ∆Ct Dose 0 (CON) − ∆Ct Dose 50, 200 or 500)) ± standard error of the mean (SEM). Positive and negative ∆∆Ct indicates upregulation and downregulation, respectively. Significance declared at *P* value ≤ 0.05 (*) and (#) denotes a statistical tendency at 0.05 < *P* value ≤ 0.10.

Cells were harvested after a 24-h incubation with various 5-HTP doses. Gene expression was analyzed and differentially expressed genes are reported as the ∆∆Ct with 5-HTP doses 50, 200 and 500 μM relative to 5-HTP 0 μM dose (Fig. 4). Refer to Supplemental Table 3 for a full list of all significant genes for the main effect of 5-HTP dose. Serotonin receptor, *5-HTR7,* was downregulated by the 200 μM dose (*P* = 0.01). Serotonin signaling genes, *AADDC* and *ALDH2* were downregulated by the 50 μM dose (*P* = 0.007) and tended to be downregulated by the 200 μM dose (*P* = 0.10). Apoptosis and autophagy related genes (i.e., *BAX, BCL2, AIFM1* and *ATG5*) were downregulated by the 200 μM dose (*P* < 0.04). Extra cellular matrix remodeling genes, *MMP14* and *MMP2*, were downregulated by the 200 μM dose and tended to be upregulated by the 50 μM dose, respectively (*P* = 0.004, 0.07). Heat shock related gene, *HSF1*, was downregulated by the 200 μM dose and tended to be downregulated by the 500 μM dose, respectively (*P* = 0.01, 0.10). Tight junction related gene, *TJP1*, tended to be downregulated by the 500 μM dose (*P* = 0.06).

Cell proliferation and survival genes measured at 24-h were impacted by 5-HTP dose. The 50 μM dose tended to downregulate and the 500 μM dose downregulated *CDNK1B* (*P* = 0.10, 0.02). The 500 μM dose tended to downregulate *CCND1* (*P* = 0.09). The 200 and 500 μM dose downregulated *PCNA* (*P* < 0.02). The 200 μM dose downregulated *AKT2* and *RHEB* (*P* < 0.02) and tended to downregulate *PIK3R2* (*P* = 0.10). The 50 μM dose tended to downregulate *MAPK10* (*P* = 0.07), while the 200 μM dose downregulated *MAPK10* (*P* = 0.01). The 200 and 500 μM dose tended to downregulate *RHOA* (*P* < 0.10).

### Interaction between temperature and 5-HTP dose

Twenty-one genes were impacted by the interaction between 5-HTP dose and temperature. These genes are displayed in Supplemental Table 4 and reported as the ∆∆Ct relative to TN-0 μM dose.

Following an 8-h incubation, HS-50 μM dose downregulated the expression of 5-HT receptor *5-HTR2C* (*P* = 0.002). Also, at 8-h, cell proliferation/survival gene *PCNA* was upregulated by TN-200, -500 μM dose and HS-50 and -500 μM dose (*P* < 0.05).

**Figure 5:**
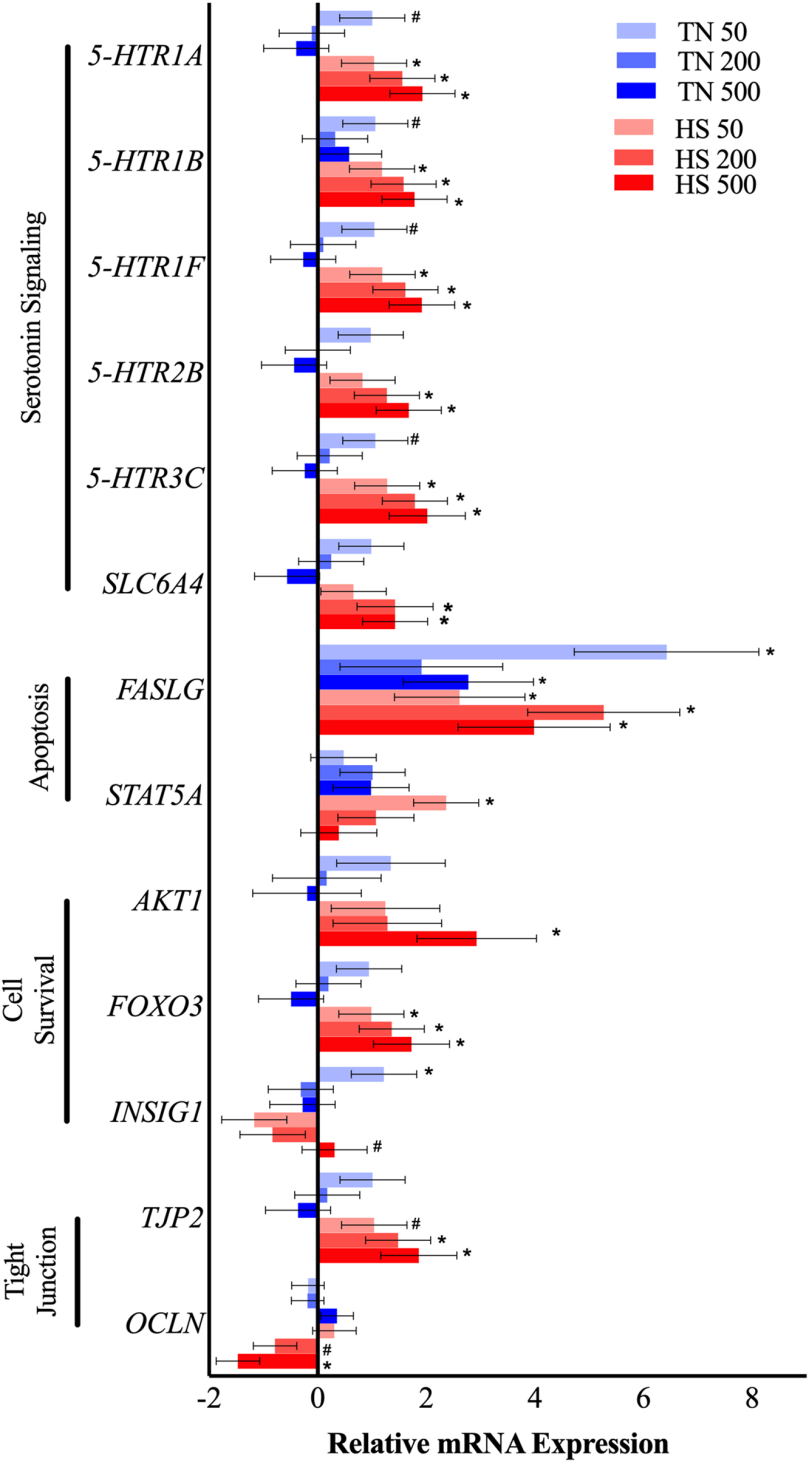
Interaction between 5-Hydroxy-L-tryptophan (5-HTP) dose and incubation temperature. Gene expression of primary bovine mammary epithelial cells (pBMEC) after 12-h exposure of either 0, 50, 200 or 500 μM dose of 5-HTP under thermoneutral (37 °C, TN) or heat shock (41.5 °C, HS) culture conditions. Data is presented as relative mRNA expression (∆∆Ct = ∆Ct TN Dose 0 (CON) − ∆Ct treatment)) ± standard error of the mean (SEM). Significance declared at *P* value ≤ 0.05 (*) and (#) denotes a statistical tendency at 0.05 < *P* value ≤ 0.10.

After a 12-h incubation (Fig. 5), 5-HT receptors *5-HTR1A, 5-HTR1B, 5-HTR1F, 5-HTR3C,* tended to be upregulated by TN-50 μM dose (*P* < 0.10) and upregulated by HS-50, -200, -500 μM dose (*P* < 0.05). Serotonin receptor, *5-HTR2B,* and the 5-HT transporter, *SLC6A4,* were both upregulated by HS-200, -500 μM dose (*P* < 0.04). Involution biomarkers, *FASLG*, was upregulated by TN-50, -500 μM dose and by HS-50, -200, -500 μM dose (*P* < 0.05), while *STAT5A* was only upregulated by HS-50 μM dose (*P* = 0.001). Autophagy related gene, *ATG5*, was downregulated by the HS-200 μM dose (*P* < 0.001) and MAPK14 tended to be downregulated by HS-500 μM dose (*P* = 0.09). Cell proliferation related gene, *AKT1*, was upregulated by HS-500 μM dose (*P* = 0.006) and *FOXO3* was upregulated by HS-200 and -500 μM dose (*P* < 0.03). Metabolic regulator, *INSIG1*, was upregulated by TN-50 μM dose (*P* = 0.05) and tended to be upregulated by HS-50 μM dose (*P* = 0.06). Tight junction gene, *TJP2*, tended to be upregulated by HS-50 μM dose (*P* = 0.10) and upregulated by HS-200 and -500 μM dose (*P* < 0.02). Another tight junction gene, *OCLN*, tended to be downregulated by HS-200 μM dose (*P* = 0.08) and downregulated by HS-500 μM dose (*P* = 0.002).

After 24-h of incubation, autophagy related gene, *ATG3*, tended to be downregulated by the TN-50 μM dose (*P* = 0.07) and cell proliferation related gene, *IGF1R*, was upregulated by TN-500 μM dose (*P* = 0.04).

### Mammary epithelial cell morphology

**Figure 6:**
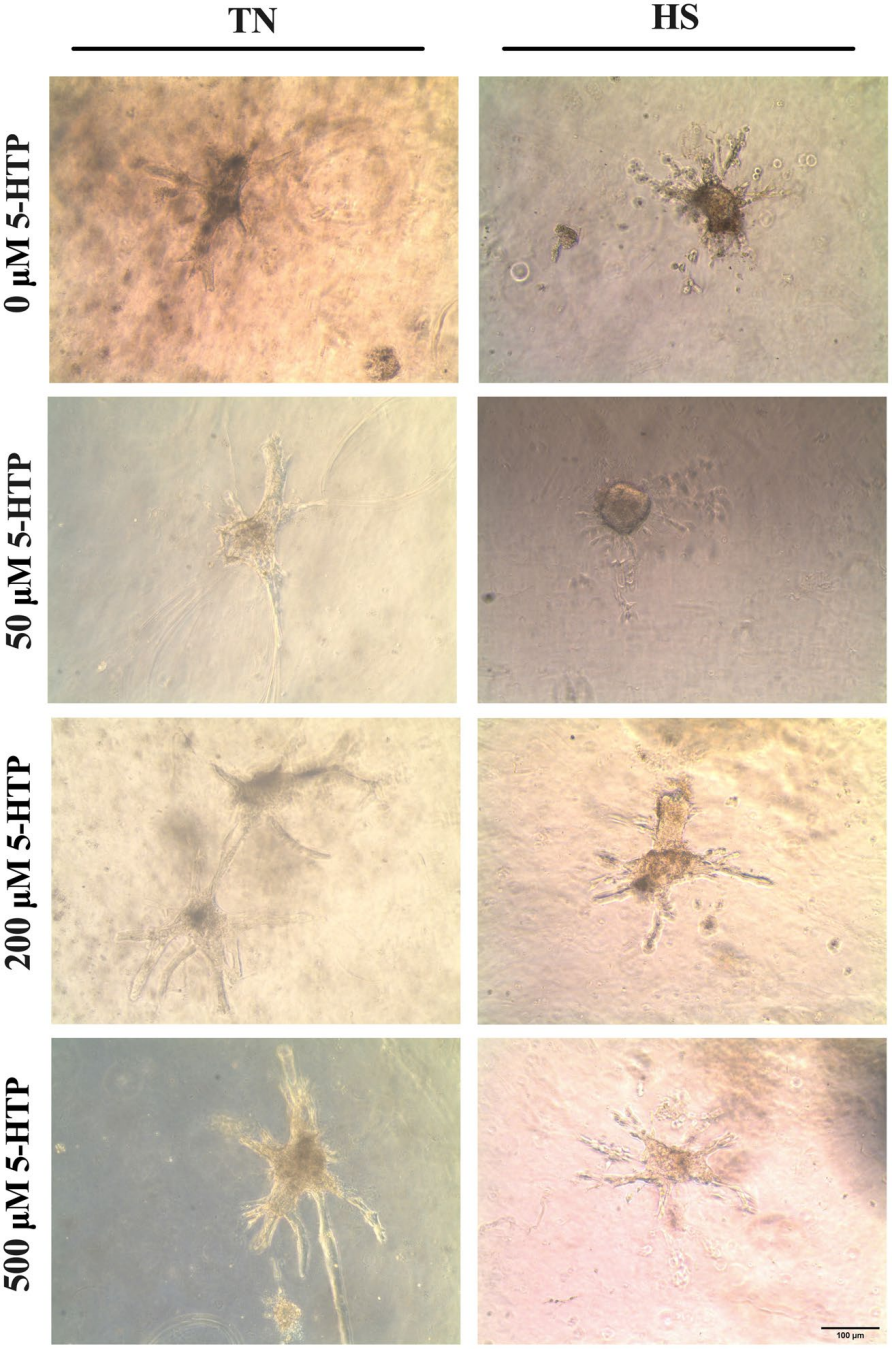
Photomicrographs of primary bovine mammary epithelial cells (pBMEC) on collagen gel cultures. Following a 24-h exposure to 0, 50, 200 or 500 μM dose of 5-Hydroxy-L-tryptophan (5-HTP) at either thermoneutral (37 °C, TN) or heat shock (41.5 °C, HS) with 5% CO_2_ culture condition. Photomicrographs were taken to visualize and quantify branch number and morphology (i.e., branch diameter and length). Scale bar indicates 100 μm.

After the 24-h incubation, photomicrographs of pBMEC were taken to visualize and quantify branch number and morphology, including branch length and diameter. pBMEC branch diameter and branch number were not different (*P* < 0.9) between 5-HTP doses or temperature conditions; however, pBMEC branch length was reduced by HS (*P* = 0.008) relative to pBMEC cultured under TN conditions (Fig. 6).

## Discussion

Serotonin acts as an autocrine-paracrine factor within the mammary gland to regulate homeostasis and trigger involution processes after milk stasis^10,17,21^. Serotonin acts in a biphasic manner in human MEC, where high concentrations and longer incubation times elicit a decrease in transepithelial electrical resistance and disruption in tight junctions; while at low concentrations and shorter incubation times 5-HT increased MEC transmembrane resistance^21^. Most research linking 5-HT and mammary function have been performed in rodent models and human cells, yet this highly conserved monoamine has been shown to be present and actively involved in the bovine mammary gland^9,10,22^. The global increase in ambient temperature imposes a threat to livestock production, including dairy cattle. More specifically, heat stress during the dry period has been shown to compromise subsequent milk yield of dairy cows^23^. This reduction has been attributed in part to a dysregulation of key genes and pathways regulating cellular events that take place during this non-lactating phase of the lactation cycle^14,15^.

Cellular exposure to heat shock exerts morphological changes including cytoskeleton remodeling, alterations in cell membrane permeability, inhibition of protein synthesis and a decrease in cell proliferation^24^. Herein, we report dynamic changes in gene expression following heat shock exposure using an in vitro cell culture model. The observed transcriptomic signature of acute heat shock cell exposure was not surprising, with the overall pattern of gene expression being consistent with previous reports^25,26^. The mRNA expression of *HSPA1A*, a heat shock protein, was dynamic, being downregulated after 8-h of HS, and upregulated after 12-h of HS. Heat shock proteins are ubiquitous, highly conserved proteins which function to inhibit or aid in apoptosis^27^. Collier and colleagues reported the heat shock protein HSP70, encoded by *HSPA1A*, had elevated expression after 4-h at 42 °C and returned to basal levels after 8-h, which this transient elevation in turn triggered the expression of apoptotic-related genes^26^. The discrepancy with previous reports could be due in part to variations in the severity and duration of the imposed heat shock (i.e., 41.5 °C vs. 42 °C), which may explain the delayed gene expression responses. An increase in *HSPA1A* gene expression in BMEC exposed to heat shock has also been previously reported^25^. These authors also report an enrichment in KEGG pathways with a downregulation in *regulation of actin cytoskeleton* and *TGF-β signaling*^25^. In our study, 8-h of HS downregulated nine out of twelve significant apoptosis-related genes measured, including *TGFB1, CASP3, BAX, BCL2, NFKB1*, and *SOCS3*. After 12-h of HS, ten out of the twelve significant apoptosis-related genes were upregulated suggesting an activation of apoptosis. Both *FAS* and *IGFBP3* are upregulated across the 8- and 12-h incubations. The *FAS* gene acts to present molecular instructions on creating proteins to initiate a signaling process known as the caspase cascade, which acts in a series of steps to initiate apoptosis. Similar reports have revealed an upregulation in *FAS* expression following a heat treatment of 42 °C in Jurkat cells, and researchers suggest HSF1 acts as an important transcription factor of the *FAS* gene^28,29^. Interestingly, 12-h of HS downregulated the expression of *HSF1.* We speculate this pattern in expression in response to HS, might activate intracellular signaling pathways to trigger cell death. *IGFBP3* is also upregulated across the 8- and 12-h incubations. Insulin-like growth factor binding protein-3 (IGFBP3) acts to facilitate the delivery of IGFs to its cell receptor, and also interacts with intracellular proteins of the ECM such as collagen and fibrin^30^. It is possible that *IGFBP3* is assisting in ECM remodeling, as indicated by the reduction in pBMEC branch length by HS.

Heat shock proteins also play a role in regulating molecular processes in the degradation and assembly of the ECM^31^. It is well known that MEC morphology and cell integrity are severely damaged by hyperthermic conditions^26,32^. Specifically, the effects of acute heat shock have previously been reported to repress ductal branching, inhibit cell proliferation, impair cell viability, and induce a greater proportion of apoptotic cells^25,26,33^. In the present study, ECM remodeling genes were downregulated after 12-h of HS, despite the orchestrated upregulation of apoptotic genes, cell survival genes, and heat shock genes. It is noteworthy that after 24-h of HS, most upregulated genes returned to expression levels of that of TN conditions. It is possible that the overall transcription pattern observed in our data set is reflecting a culmination of acute thermoregulatory mechanisms after prolonged severe heat shock.

Following our objective to explore the response of acute heat shock on the expression of genes related to 5-HT synthesis, signaling and metabolism, we supplemented incremental concentrations of 5-HTP to the media. Out of the 14 5-HT receptors evaluated herein, the mRNA expression of the isoforms -*2A* and -*3B* were not detected in pBMEC at any timepoint. The serotonin molecule is unique in which it can bind to seven receptor families, which total to more than twelve subtypes. Serotonin receptors primarily consists as G-Protein coupled receptors (GPCR) and once ligand-bound, can activate G_s_, G_q/11_, or G_i/o_ intracellular subunits to activate a variety of signaling cascades to modulate the expression and activity of gene transcription and proteins^34^. Herein, we observed a consistent downregulation after 8-h HS incubation of 5-HT synthesis and metabolism genes (including 5-HT receptors and associated downstream signaling, and 5-HT degradation genes). This expression pattern shifted after 12-h, when most serotonin-related genes were upregulated, then returning to TN expression levels after 24-h of exposure. The dynamic pattern of gene expression in response to heat shock is similar to that observed for apoptosis related genes (i.e., *CASP3, BAX* and *BCL2*), which might be viewed as a concerted attempt to restore cellular homeostasis after the initial HS insult. After 8-h HS, *5-HTR7* was downregulated, as well as tight junction genes *TJP2, CLDN3, -4, -5*, and *-7*. The 5-HTR7 is coupled to adenyl cyclase through the G_s_ subunit, through cAMP and is known to regulate tight junction status. It is possible that the downregulation in 5-HTR7 gene expression could drive the downregulation in tight junction gene expression observed at this timepoint. Literature investigating the role of 5-HT in heat stress responses is limited, although our laboratory recently reported lower circulating serotonin concentrations in dairy calves exposed to chronic heat stress^35^. The stress kinase p38-MAP kinase is the major intracellular signal cascade activated by heat shock^36^. Interestingly, p38-MAP kinase activators in the presynaptic membrane revealed an elevation in 5-HT transport through SERT^37^. The changes in 5-HT receptor expression seen from HS could be modulated by the intracellular kinases activated through the heat shock response. However, there is currently a gap in knowledge of which receptor subtypes that modulate which specific physiological responses within the mammary gland. The mammary serotonergic system plays a role in lactation homeostasis; therefore, the negative effects seen by heat shock could be due in part from impaired 5-HT signaling via its receptors in the mammary gland. This data warrants further investigation to uncover the mechanisms underlying the heat shock response and serotonin’s role in this highly regulated process.

Maintenance of tight junction permeability between epithelial cells is crucial to regulate the unidirectional movement of cell secretions and components through the paracellular pathway^38^. Mammary epithelial cell tight junctions are regulated by endocrine and paracrine signals, including 5-HT. The preservation and maintenance of tight junctions is essential for a successful lactation. Serotonin triggers the opening of tight junctions which become leaky, and is a hallmark of mammary gland involution^9^. Specifically, 5-HT dissipates the transepithelial gradients necessary for milk secretion and increases tight junction permeability, acting as a feedback inhibitor of lactation^9,20,39^. In the present study, after 24-h of heat shock, the 500 μM 5-HTP dose tended to downregulate the mRNA expression of *TJP1* and the 200 μM 5-HTP dose downregulated the expression of *5-HTR7*. In human and mouse MEC cultured in vitro, 5-HTR7 has been associated with tight junction regulation. MEC transepithelial resistance is mediated by 5-HTR7 which acts through the G_s_/cAMP intracellular pathway. Serotonin binding to 5-HTR7 in MEC couples to the G_s_ subunit, which in turns activates the signal transduction pathway to increase levels of cAMP, which is thought to regulate tight junction activity^9^.

Tight junctions are a multiprotein complex, orchestrated to regulate the flow of molecules to the intracellular space between epithelial cells. Occludin, claudins, and zonula occludens (also known as tight junction proteins 1, -2 and -3) together regulate the function, formation, and maintenance of tight junctions^40^. Insights into the exact role of these proteins and their activity in regulating tight junction permeability is beginning to emerge. After 8- and 12-h of HS, the gene expression of *TJP2* and *TJP3* was downregulated. However, after 12-h of HS with 200 and 500 μM 5-HTP, *TJP2* gene expression was upregulated. Furthermore, after 12-h of HS, *OCLN* was upregulated, then with 200 and 500 μM 5-HTP at HS conditions *OCLN* expression was downregulated at 12-h. It is suggested the addition of 5-HTP, in the presence of HS, assists in the downregulation of *OCLN*, which further supports serotonin’s role in decreasing tight junction permeability. Interestingly, 5-HT has been shown to disrupt tight junction permeability in tissues other than the mammary gland. In human esophageal epithelial cells, 5-HT reduced the protein expression of OCLN, TJP1 and induced the phosphorylation of p38 MAP kinases^41^. Serotonin signaling pathways within the mammary gland may mediate the molecular processes involved in the breakdown of tight junctions during involution in dairy cows experiencing hyperthermia.

Microstructural and cellular changes during mammary involution include increased MEC death and remodeling of tissue architecture. Matrix Metalloproteinases (MMP) primarily interact in the stromal cells to break-down of the ECM and assist in tissue remodeling^42^. In the mammary gland, several MMPs subtypes have been identified acting through different substrates. Herein, following a 24-h incubation with 5-HTP, *MMP14* and *MMP2*, were downregulated by the 200 μM 5-HTP dose and tended to be upregulated by the 50 μM 5-HTP dose, respectively. Induced heart attack in 5-HTT (serotonin transporter) knock out models displayed an upregulated expression of MMP2 in the heart^43^. These authors postulate the 5-HT deficiency to be responsible for an impaired healing of the heart muscle. Matrix Metalloproteinase-2 also acts to facilitate wound healing recovery in the central nervous system; therefore, a downregulation could suggest a more active breakdown of matrix and non-matrix proteins in MEC^44^. Interestingly, stimulation of 5-HTR7 by receptor agonist 5-carboxamidotryptamine maleate has been shown to directly increase MMP-9 expression with a subsequent increase in dendritic spine remodeling^45^. The exact mechanisms by which 5-HT modulates MMP gene expression in the mammary gland is unknown, although it appears to be dose and subtype specific. It is tempting to speculate that promoting local 5-HT synthesis within the bovine MEC might modulate MMPs expression which might, in turn, assist with ECM breakdown resulting in a more efficient and rapid involution process. Incremental 5-HTP doses did not modulate the expression of genes when assessed after only 8-h of incubation. It takes more than 8 h to significantly increase intracellular serotonin concentrations to exert molecular actions within MEC. This observation in supported by the fact that differential gene expression is observed only at 12- and 24-h. Similar reports in mouse MEC reported no increase in intracellular 5-HT concentrations with 500 μM 5-HTP until 12-h incubation^46^. The timing of events for serotonin signaling within the cell is crucial to delineate its effects in in vitro studies.

The mechanistic link between 5-HT actions under heat shock conditions within MEC are beginning to be elucidated. Yet, there is more to understand about the potential role of 5-HT under heat shock at the cellular level. Herein, we characterized the gene expression profile of genes related to 5-HT signaling and key cellular processes taking place during mammary gland involution that are critical for a successful lactation under heat shock and incremental 5-HTP doses combinations. Of interest, *5HTR-1A, -1B, -1F* were upregulated by 200 and 500 μM 5-HTP under HS conditions. Previous literature has reported an upregulation in *5-HTR1A* and *-1F* in circulating leukocytes in dairy calves exposed to chronic heat stress^35^. Although data is limited in the bovine, research in other species and experimental models is available. For instance, in nematodes, excitation of thermosensory and serotonergic neurons activated the heat shock response through the increase in 5-HT release^47^. Further, utilizing *TPH1* KO nematodes it was shown that 5-HT was released in response to temperature increases in an AFD thermosensory neuron-dependent manner^47^. These findings demonstrate that 5-HT is a potential regulator of the heat shock response. Our data indicates that 5-HT synthesis, signaling, and metabolism in pBMEC is triggered by exogenous addition of 5-HTP to the media. However, the implication of these findings warrants further investigation since only gene expression levels are presented.

Cell turn over during the dry period is primarily characterized by MEC growth and death, primarily by cell proliferation and apoptosis/autophagy which are key cellular processes taking place at different rates throughout the length of the dry period in dairy cattle. The efficient removal of senescent MEC (i.e., by apoptosis) and the concomitant rise in MEC number (i.e., by cell proliferation) is crucial for a successful subsequent lactation. These cellular processes are sensitive and greatly impacted by hyperthermia^26,48^. Previous studies have also reported, heat stress during the early dry period reduces MEC apoptosis and proliferation, leading to a disruption in mammary gland cellular turnover, therefore affecting the secretory capacity in the next lactation^39^. Serotonin has been shown to increase cell proliferation within the mammary gland of rodents. Specifically, whole body *TPH1* KO mice have a sixfold decrease in cell proliferation within the mammary gland, and administration of exogenous 5-HTP restored cell proliferation to wild-type levels^22^. In the present study, *AKT1* was upregulated in pBMEC cultured under HS-500 μM 5-HTP. This might indicate that serotonin induces cell survival mechanisms through an activation of intracellular pathways downstream within the cell when under hyperthermic stress, which could suggest synergism of high 5-HTP concentrations with heat shock in MEC.

The communication between intracellular proteins within the MEC is vastly complex. Studies suggest pAKT acts as a molecular guard for death and survival signals within MEC^49^. A transgenic mouse model revealed that constitutively active AKT (Protein Kinase B) in the mammary gland resulted in a delayed onset of apoptosis during involution^50^. Serotonin binding to 5-HTR4, -6, and -7 receptors can activate the downstream PI3K-AKT signaling pathway, in turn, regulating cell proliferation and apoptosis. Under heat shock, serotonin’s role seems to be compensatory by upregulating cell proliferation genes. Interestingly, *FASLG* gene expression was upregulated by all treatments except TN-200. Fas ligand (FASLG), is a death receptor ligand participating in the regulation of apoptosis in the involuting mammary gland, in part through transcriptional regulation^49^. Herein, pBMEC cultured under heat shock combined with various 5-HTP doses resulted in a significant upregulation in apoptotic genes in pBMEC. The Signal Transducer and Activator of Transcription 5a (*STAT5a*) mRNA was upregulated in pBMEC under HS cultured with 50 μM of 5-HTP. The STAT5a intracellular protein plays a prominent role in lobuloalveolar development^12,51^. We speculate that the modulation of the gene expression of these key intracellular proteins by serotonin, coupled with an upregulation in cell proliferation genes, might indicate a role in accelerating cellular turnover during involution. Future studies assessing the translational profile of intracellular proteins downstream of serotonin receptors and of apoptotic and proliferative protein biomarkers and well as the localization of tight junction proteins within the cell are necessary.

## Conclusions

Consistent with previous reports, there was a potent effect of HS on pBMEC gene expression, particularly after 8- and 12-h of exposure. Incremental doses of 5-HTP alone modulated pBMEC gene expression only after 12- and 24-h of incubation. Addition of exogenous 5-HTP to pBMEC cultured under HS conditions modulated the expression of genes related to cell-turn over, cell survival and tight junctions possibly through its downstream actions on key serotonin receptors families, indicated by an orchestrated upregulation in gene expression. Addition of exogenous 5-HTP to MEC under HS in vitro modulates the expression of key genes involved in cellular turnover and tight junction breakdown, which might aid in a more effective and rapid involution processes. It is important to note that the current study characterized changes in cell morphology and transcriptomic profile of 96 genes within 9 pathways of interest under various treatment conditions. Therefore, results should be interpreted with caution and the impact on protein expression should be further explored. Additionally, in vivo studies targeting the serotonergic mammary system in dairy cattle experiencing hyperthermia are needed to determine the potential of this molecule to accelerate MEC turn over during involution.

## Methods

Cows were humanely euthanized at an abattoir where tissue dissociation and primary epithelial cell isolation were performed according to procedures described^26,52^. Briefly, 10 g of minced parenchyma tissue was dissociated using 100 mL of dissociation medium consisting of 0.15% collagenase type II, 0.075% hyaluronidase, and 5% fetal bovine serum while continuously shaken. The dissociated tissue was filtered three times using Nitex mesh filter paper, then all cell isolates were resuspended in Medium 199 and 10% fetal bovine serum. Cells were isolated from the mammary gland of three multiparous, pregnant nonlactating Holstein dairy cows immediately following euthanasia and cell vials were stored in liquid nitrogen until vials were randomly selected for this study. The data presented from our study did not involve any manipulation of live cows. Approximately 1 × 10^7^ cells/mL were plated in a 24-well dish on a rat tail collagen matrix and sustained in media consisting of DMEM/F-12 + 10 mM acetate, 10 μg/mL insulin (#10516, Sigma-Aldrich/MDS), 25 ng/mL recombinant-human epidermal growth factor (EGF, #13247-051, Invitrogen Corp.), 1:100 antibiotic/antimycotic (100 U/mL penicillin, 100 μg/mL streptomycin and 0.25 μg amphotericin B; #15240, Invitrogen Corp., Carlsbad, CA), 0.1% bovine serum albumin and 5 ng/mL progesterone to induce growth, proliferation and ductal development. The collagen base layer reagents and protocol can be found in Hernandez et al.^20^. The growth media was changed every 48 h for 7 d until cells reached confluency (i.e., pBMEC were visualized at 20X to confirm branch maturity). On d 8, four concentrations of 5-HTP (#4350-09-8, Sigma-Aldrich) were applied to the corresponding wells. Increasing doses of 5-HTP were added to the proliferation media (0, 50, 200 or 500 μM) and incubated at either thermoneutral (TN, 37 °C) or heat shock (HS, 41.5 °C) conditions for 8, 12, or 24-h with 5% CO_2_. Each plate contained 6 wells of each 5-HTP dose which were combined into three technical replicates (Supplemental Fig. 1) and the experiment was repeated three times (i.e., 3 blocks) for three biological replicates. Treatment and incubation time were halted at 0, 8-, 12- or 24-h. Cells were harvested with 500 μl TRIzol Reagent (Invitrogen, cat # 15596026) per well. Two wells were combined and stored at − 80 °C until RNA extraction. RNA was extracted by adding 200 μl of chloroform for precipitation following TRIzol manufacturer’s instructions. Pellets were allowed to dry 5–10 min, then resuspended in 25 μl ddH_2_0. The RNA concentration and integrity were assessed using a NanoDrop (#ND-2000, Thermo Scientific, Wilmington, DE). RNA samples were stored at -80 °C until gene expression analysis.

To evaluate the expression of genes associated with serotonin signaling and metabolism (i.e., receptors and downstream intracellular pathways, synthesis, uptake, and degradation), involution biomarkers (i.e., genes associated with cellular processes such as apoptosis, autophagy, cell proliferation and survival), tight junction genes, and ECM remodeling genes, a high-throughput Multiplex RT-qPCR BioMark Dynamic Array Integrated Fluidic Circuits (IFCs) was utilized (Fluidigm Corporation, South San Francisco, CA). Briefly, ninety-six primers targeting 92 genes of interest, 3 reference genes (*β-actin, HPRT-1* and *RSP9*) and one structural reference gene were assayed (Supplemental Table 1). As previously described by Marrero et al.^53^, samples were normalized to 256 pg RNA and transferred to the IFC plate with the appropriate primer–probe sets. All reactions were performed following the manufacturer’s thermal protocol: 95 °C for 1 min, followed by 30 cycles at 96 °C for 5 s and 60 °C for 20 s. The Fluidigm Real-Time PCR Analysis software was utilized to calculate Ct values for all 96 genes for all samples analyzed. Primary BMEC non-detectable expression was set at a Ct of 28.9. The NormFinder and BestKeeper software were used to determine the most stable housekeeping gene across time, 5-HTP dose and temperature for all samples. The mRNA expression of *RSP9* was determined as suitable housekeeping gene and was used to normalize Ct values. The normalized gene expression (∆Ct) was used for statistical analysis. After a 24-h incubation, photomicrographs of pBMEC were taken with a Zeiss Axio Vert. A1 microscope at 20 X magnification, to visualize branch number and morphology. Branch number, length and diameter were determined using Image J software^54^. Data were analyzed by analysis of variance using the MIXED procedure of SAS (version 9.4, SAS Institute, Inc., Cary, NC, USA). Normalized gene expression (∆Ct) at each of the three harvesting times (8, 12 and 24 h) was analyzed separately. The model included 5-HTP dose (0, 50, 200, 500 µM), temperature (TN vs. HS) and their interaction (dose*temperature) as fixed effects, and block as random effect. Residuals were tested for normality and homogeneity of variance. Gene expression data is presented as the estimates of the model (i.e., ∆∆Ct). Statistical differences were made by Dunnett testing and computed as HS relative to TN for the temperature, all doses relative to 0 μM for 5-HTP for the dose, and the interaction relative to TN-0 μM. Statistical significance was declared at *P* ≤ 0.05 and *P* ≤ 0.10 were considered a tendency towards significance.

## Supplementary Information

Supplementary Information.
