# Supplementary material for: In vitro effects of 5-Hydroxy-L-tryptophan supplementation on primary bovine mammary epithelial cell gene expression under thermoneutral or heat shock conditions
Source: Sci Rep. 2022 Mar 9;12:3820. doi: 10.1038/s41598-022-07682-7 (PMC8907223; doi:10.1038/s41598-022-07682-7)
Supplement: Supplementary file 1 — Supplementary Information. [file 41598_2022_7682_MOESM1_ESM.docx]

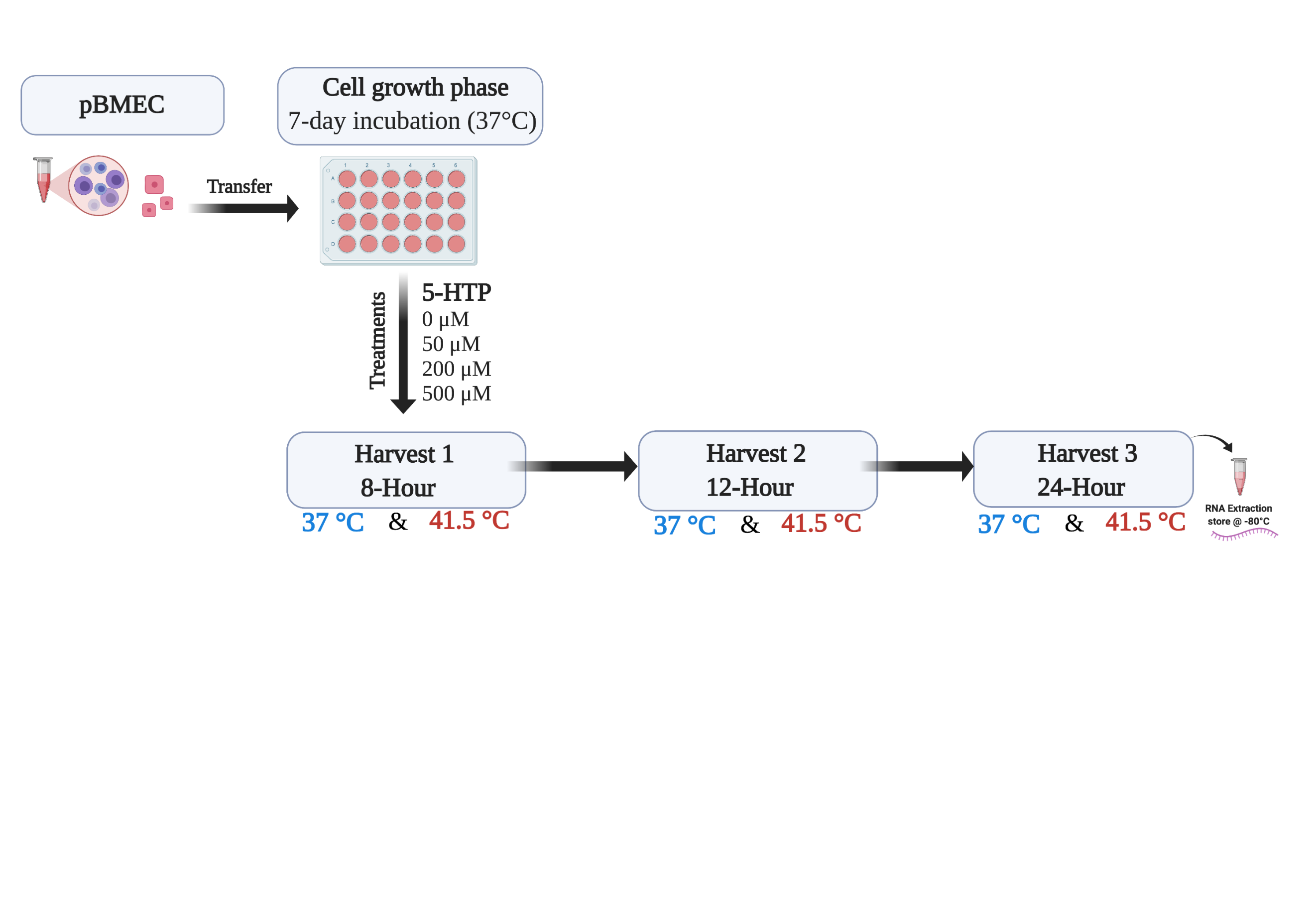


**Supplementary Figure 1**

Experimental timeline of events displaying primary bovine mammary epithelial cells (pBMEC) growth phase and treatment assignment to plate wells. Four concentrations of 5-Hydroxy-L-tryptophan (5-HTP; 0, 50, 200 or 500 μM)) were added to the proliferation media, as indicated in Hernandez et al., (2008)^20^, and incubated at either thermoneutral (TN, 37°C) or heat shock (HS, 41.5°C) conditions for 8, 12, or 24 hours. Each harvest was followed by RNA extraction, which was then stored in -80°C until further gene expression analysis. The experiment was repeated three times.

**Supplementary Table 1.** Gene symbols and name of 95 genes tested for Bos Taurus.

|  |  | Gene |  | Gene Name |
| --- | --- | --- | --- | --- |
| 1 |  | *ACTB* |  | Beta Actin |
| 2 |  | *HPRT-1* |  | Hypoxanthine Phosphoribosyltransferase-1 |
| 3 |  | *RSP9* |  | Ribosomal Protein-9 |
| 4 |  | *5-HT1A* |  | Serotonin Receptor 1A |
| 5 |  | *5-HT1B* |  | Serotonin Receptor 1B |
| 6 |  | *5-HT1D* |  | Serotonin Receptor 1D |
| 7 |  | *5-HT1F* |  | Serotonin Receptor 1F |
| 8 |  | *5-HT2A* |  | Serotonin Receptor 2A |
| 9 |  | *5-HT2B* |  | Serotonin Receptor 2B |
| 10 |  | *5-HT2C* |  | Serotonin Receptor 2C |
| 11 |  | *5-HT3A* |  | Serotonin Receptor 3A |
| 12 |  | *5-HT3B* |  | Serotonin Receptor 3B |
| 13 |  | *5-HT3C* |  | Serotonin Receptor 3C |
| 14 |  | *5-HT4* |  | Serotonin Receptor 4 |
| 15 |  | *5-HT5a* |  | Serotonin Receptor 5A |
| 16 |  | *5-HT6* |  | Serotonin Receptor 6 |
| 17 |  | *5-HT7* |  | Serotonin Receptor 7 |
| 18 |  | *AADC* |  | Aromatic L-amino acid decarboxylase |
| 19 |  | *ACTL8* |  | Actin Like 8 |
| 20 |  | *AGT* |  | Angiotensinogen |
| 21 |  | *AIFM1* |  | Apoptosis inducing factor mitochondria associated 1 |
| 22 |  | *AKT1* |  | serine/threonine kinase 1 |
| 23 |  | *AKT2* |  | Protein kinase B (PKB) or AKT |
| 24 |  | *ALDH2* |  | alcohol dehydrogenase (ADH)2 |
| 25 |  | *APAF1* |  | apoptic peptidase activating factor 1 |
| 26 |  | *ATG3* |  | Autophagy related ATG3 |
| 27 |  | *ATG5* |  | Autophagy related ATG5 |
| 28 |  | *ATG7* |  | Autophagy related 7 ATG7 |
| 29 |  | *BACH2* |  | BTB domain and CNC homolog 2 |
| 30 |  | *BAD* |  | BCL2 associated agonist of cell death |
| 31 |  | *BAX* |  | BCL2 associated X / bcl-2-like protein 4 |
| 32 |  | *BCL2* |  | apoptosis regulator BCL2 |
| 33 |  | *CASP3* |  | Caspase 3 |
| 34 |  | *CASP8* |  | Caspase 8 |
| 35 |  | *CASP9* |  | Caspase 9 |
| 36 |  | *CCND1* |  | Cyclin D1 |
| 37 |  | *CDKN1B* |  | Cyclin dependent kinase inhibitor 1B |
| 38 |  | *CLDN1* |  | Claudin 1 |
| 39 |  | *CLDN3* |  | Claudin 3 |
| 40 |  | *CLDN4* |  | Claudin 4 |
| 41 |  | *CLDN5* |  | Claudin 5 |
| 42 |  | *CLDN7* |  | Claudin 7 |
| 43 |  | *CXCL2* |  | Chemokine ligand 2 |
| 44 |  | *DACT2* |  | Disheveled binding antagonist of beta catenin 2 |
| 45 |  | *DNAJC12* |  | Heat shock protein-40 |
| 46 |  | *EGF* |  | epidermal growth factor |
| 47 |  | *FAS* |  | Fas cell surface death receptor |
| 48 |  | *FASLG* |  | Fas-Ligand |
| 49 |  | *FOXO3* |  | Forkhead Box |
| 50 |  | *GADD45A* |  | growth arrest & DNA damage inducible alpha |
| 51 |  | *HSF1* |  | Heat shock transcription factor 1 |
| 52 |  | *HSP90AA1* |  | Heat shock protein family A HSP90 |
| 53 |  | *HSPA1A* |  | Heat shock protein family A member 1A (HSP70) |
| 54 |  | *HSPD1* |  | HSP Family D Member 1 (HSP60) |
| 55 |  | *IDO1* |  | Indoleamine 2,3-dioxygenase |
| 56 |  | *IGF1* |  | Insulin like growth factor 1 |
| 57 |  | *IGF1R* |  | Insulin growth factor receptor |
| 58 |  | *IGFBP3* |  | Insulin like growth factor binding protein 3 |
| 59 |  | *IGFBP5* |  | Insulin like growth factor binding protein 5 |
| 60 |  | *INSIG1* |  | insulin induced gene 1 |
| 61 |  | *LCA5L* |  | Lebercilin LCA5 like |
| 62 |  | *MAOA* |  | Monoamine oxidase A |
| 63 |  | *MAOB* |  | Monoamine oxidase B |
| 64 |  | *MAPK10* |  | mitogen-activated protein kinase 10 |
| 65 |  | *MAPK14* |  | Mitogen-activated protein kinase 14 |
| 66 |  | *MAPK3* |  | Same as ERK1 |
| 67 |  | *MMP1* |  | Matrix Metallopeptidase 1 |
| 68 |  | *MMP14* |  | Matrix Metallopeptidase 14 |
| 69 |  | *MMP2* |  | Matrix Metallopeptidase 2 |
| 70 |  | *mTOR* |  | Mechanistic target of rapamycin (MTOR) |
| 71 |  | *NFKB1* |  | nuclear factor kappa B subunit 1 |
| 72 |  | *OCLN* |  | Occludin |
| 73 |  | *PCNA* |  | Proliferating Cell Nuclear Antigen |
| 74 |  | *PIK3CB* |  | Phosphatidyl inositol Catalytic Subunit beta |
| 75 |  | *PIK3R2* |  | Phosphoinositide-3-kinase regulatory subunit 2 |
| 76 |  | *PRKACA* |  | activated catalytic subunit alpha |
| 77 |  | *PTGES* |  | Prostaglandin E synthase |
| 78 |  | *RAC1* |  | Rac family small GTPase |
| 79 |  | *RHEB* |  | Ras homolog mTOR1 binding |
| 80 |  | *RHOA* |  | Ras homolog family member A |
| 81 |  | *SLC6A4* |  | Serotonin Transporter (SERT) |
| 82 |  | *SOCS3* |  | Suppressor of Cytokine Signaling 3 |
| 83 |  | *STAT3* |  | Signal Transducer and Activator of Transcription 3 |
| 84 |  | *STAT5A* |  | Signal Transducer and Activator of Transcription 5A |
| 85 |  | *TEKT3* |  | Tektin 3 |
| 86 |  | *TFEB* |  | Transcription factor EB |
| 87 |  | *TGFB1* |  | Transforming Growth factor Beta 1 |
| 88 |  | *TGFB1* |  | Transforming Growth Factor Beta 1 |
| 89 |  | *TJP1* |  | Tight Junction Protein 1 (Zo1) |
| 90 |  | *TJP2* |  | Tight Junction Protein 2 (Zo2) |
| 91 |  | *TJP3* |  | Tight Junction Protein 3 (Zo3) |
| 92 |  | *TNF* |  | Tumor Necrosis Factor |
| 93 |  | *TNFRSF1A* |  | Tumor Necrosis Factor Receptor Superfamily member 1A |
| 94 |  | *TPH1* |  | Tryptophan hydroxylase 1 |
| 95 |  | *WIF1* |  | WNT inhibitory factor 1 |

**Supplementary Table 2.** Gene expression of primary bovine mammary epithelial cells (pBMEC) after 8-, 12- or 24-hour exposure to either thermoneutral (37°C, TN) or heat shock (41.5°C, HS). Data is presented as ∆∆Ct (∆∆Ct = ∆Ct TN - ∆Ct HS) and standard error of the mean (SEM). Positive and negative ∆∆Ct indicates upregulation and downregulation, respectively. Significance was declared at *P*-value ≤ 0.05 (bold) and tendencies at 0.05 < *P*-value ≤ 0.10 (italicized).

|  | **TN *vs.* HS** | |  | **TN *vs.* HS** | |  | **TN *vs.* HS** | |
| --- | --- | --- | --- | --- | --- | --- | --- | --- |
|  | 8 Hour | |  | 12 Hour | |  | 24 Hour | |
| **Gene** | ∆∆Ct ± S.E | P-value |  | ∆∆Ct | P-value |  | ∆∆Ct | P-value |
| *5-HT1A* | -2.24 ± 0.79 | **<.0001** |  | 1.40± 1.25 | **<.0001** |  | -0.24 ± 0.31 | 0.495 |
| *5-HT1B* | -2.03± 0.58 | **0.0005** |  | 1.18 ± 0.65 | **<.0001** |  | 0.11 ± 0.63 | 0.749 |
| *5-HT1D* | -1.62± 0.7 | **0.0008** |  | 0.91 ± 0.63 | **0.0143** |  | 0.28 ± 0.67 | 0.471 |
| *5-HT1F* | -2.02± 0.61 | **0.0004** |  | 1.43 ± 1.35 | **<.0001** |  | -0.28 ± 0.28 | 0.420 |
| *5-HT2B* | -2.16± 0.35 | **0.0001** |  | 1.22 ± 1.59 | **0.0003** |  | -0.14 ± 0.29 | 0.688 |
| *5-HT2C* | -2.38± 0.57 | **0.0054** |  | 1.15 ± 1.63 | **0.003** |  | -0.99 ± 0.41 | *0.086* |
| *5-HT3A* | -3.82± 1.61 | **0.0095** |  | 2.91 ± 1.08 | **0.0028** |  | -0.34 ± 0.41 | 0.597 |
| *5-HT3C* | -2.11± 0.88 | **0.0002** |  | 1.52 ± 1.21 | **<.0001** |  | -0.30 ± 0.37 | 0.408 |
| *5-HT4* | -1.90± 0.65 | **0.0008** |  | 1.35 ± 1.06 | **<.0001** |  | -0.13 ± 0.52 | 0.686 |
| *5-HT5A* | -2.08± 0.79 | **0.0009** |  | 2.40± 1.01 | **<.0001** |  | 0.43 ± 1.32 | 0.318 |
| *5-HT6* | -2.38± 0.93 | **0.0025** |  | 1.89 ± 0.71 | **<.0001** |  | 0.07 ± 0.61 | 0.859 |
| *5-HT7* | -2.01± 0.92 | **0.0003** |  | 1.41 ± 1.01 | **<.0001** |  | -0.28 ± 0.54 | 0.402 |
| *AADC* | -2.12± 0.57 | **0.0138** |  | 1.20 ± 1.16 | **0.0465** |  | 0.08 ± 0.7 | 0.896 |
| *ACTL8* | -2.01± 0.59 | **0.0008** |  | 0.18 ± 0.87 | **<.0001** |  | 0.69 ± 1.2 | *0.102* |
| *AIFM1* | -1.47± 0.37 | **0.0098** |  | 0.18 ± 0.87 | 0.5831 |  | -0.38 ± 0.28 | 0.340 |
| *AKT1* | -1.73± 0.52 | **0.0227** |  | 1.59 ± 1.28 | 0.0026 |  | -0.88 ± 0.32 | *0.067* |
| *AKT2* | -0.73± 0.75 | **0.1366** |  | 0.81 ± 0.49 | 0.0074 |  | 0.01± 0.63 | 0.965 |
| *ALDH2* | 0.54± 0.57 | 0.4158 |  | -1.50 ± 0.16 | **<.0001** |  | -0.08 ± 0.38 | 0.856 |
| *APAF1* | -1.09± 0.54 | **0.1391** |  | 0.43 ± 0.44 | 0.4222 |  | -0.11 ± 0.8 | 0.866 |
| *ATG3* | 0.55± 0.46 | 0.2877 |  | -0.26 ± 0.58 | 0.5933 |  | -0.36 ± 0.33 | 0.379 |
| *ATG5* | -0.17± 0.47 | 0.7441 |  | -0.54 ± 3.13 | **0.0085** |  | 0.75 ± 0.34 | *0.083* |
| *ATG7* | -0.22± 0.31 | 0.6711 |  | -0.53 ± 0.26 | 0.0928 |  | -0.49 ± 0.24 | 0.162 |
| *BACH2* | -2.01± 0.64 | **0.0017** |  | 2.32 ± 1.4 | **<.0001** |  | 0.24 ± 1.17 | 0.611 |
| *BAD* | 0.22± 0.27 | 0.5628 |  | -0.49 ± 0.31 | **0.0035** |  | -0.008 ± 0.33 | 0.983 |
| *BAX* | -2.41± 0.64 | **0.0008** |  | 1.73 ± 1.27 | **<.0001** |  | -0.51 ± 0.31 | 0.209 |
| *BCL2* | -2.03± 0.71 | **0.0005** |  | 1.69 ± 1.18 | **<.0001** |  | 0.05 ± 0.45 | 0.888 |
| *CASP3* | -1.09± 0.51 | **0.0174** |  | 1.74 ± 0.24 | **<.0001** |  | -0.63 ± 0.75 | 0.141 |
| *CASP8* | 0.03± 0.35 | 0.9451 |  | 0.51 ± 0.65 | 0.2692 |  | 0.16 ± 0.40 | 0.694 |
| *CASP9* | -0.27± 0.31 | 0.5999 |  | 0.75 ± 0.3 | **0.0512** |  | -0.55 ± 0.71 | 0.465 |
| *CCND1* | 0.57± 0.57 | 0.3333 |  | -0.05 ± 0.78 | 0.8069 |  | -0.07 ± 0.40 | 0.858 |
| *CDKN1B* | -0.49± 0.36 | 0.3482 |  | 0.29 ± 0.4 | 0.5776 |  | -0.88 ± 0.40 | *0.075* |
| *CLDN1* | -0.60± 0.32 | 0.2757 |  | -0.66 ± 0.32 | **0.0944** |  | -1.23 ± 0.43 | **0.045** |
| *CLDN3* | -1.99± 0.5 | **0.0002** |  | 0.83 ± 0.42 | **0.0029** |  | -0.10 ± 0.71 | 0.735 |
| *CLDN4* | -1.67± 0.42 | **0.0004** |  | 0.76 ± 0.67 | **0.0097** |  | -0.36 ± 0.74 | 0.242 |
| *CLDN5* | -2.59± 0.63 | **<.0001** |  | 1.80 ± 0.55 | **<.0001** |  | 0.02 ± 0.75 | 0.938 |
| *CLDN7* | -0.99± 0.52 | **0.0253** |  | 0.22 ± 0.35 | 0.4042 |  | -0.01 ± 0.41 | 0.967 |
| *CXCL2* | -0.83± 0.45 | 0.2297 |  | 0.71 ± 0.51 | 0.2569 |  | -1.10 ± 1.16 | 0.115 |
| *DACT2* | -2.77± 1.15 | **0.0275** |  | 3.25 ± 0.64 | **0.0046** |  | -0.54 ± 0.50 | 0.459 |
| *EGF* | 2.12± 0.55 | **0.003** |  | 2.25 ± 0.38 | **0.0003** |  | 0.51 ± 1.08 | 0.248 |
| *FAS* | 1.32± 0.57 | **0.043** |  | 1.95 ± 0.35 | **<.0001** |  | -0.22 ± 0.84 | 0.617 |
| *FASLG* | 7.74± 0.97 | **0.1159** |  | - | - |  | -0.32 ± 1.51 | 0.685 |
| *FOXO3* | 0.92± 0.61 | **0.0003** |  | 1.23 ± 1.25 | **0.0003** |  | -0.36 ± 0.25 | 0.306 |
| *GADD45A* | 4.04± 0.44 | **0.0142** |  | 1.26 ± 0.97 | **0.0006** |  | 0.20 ± 0.37 | 0.663 |
| *HSF1* | 3.84± 0.45 | **0.073** |  | -0.86 ± 0.77 | **0.0068** |  | 0.46 ± 0.29 | 0.266 |
| *HSP90AA1* | -1.72± 0.46 | 0.4193 |  | 1.26 ± 1.48 | **0.0039** |  | 1.15 ± 0.57 | **0.001** |
| *HSPA1A* | -0.77± 0.69 | **0.0085** |  | 1.51 ± 0.46 | **<.0001** |  | 0.11 ± 0.63 | 0.717 |
| *HSPD1* | 1.74± 0.8 | **0.0479** |  | 0.90 ± 0.41 | **0.0002** |  | 0.29 ± 0.50 | 0.288 |
| *IDO1* | -0.16± 0.62 | **0.0004** |  | 1.42 ± 1.39 | **<.0001** |  | -0.32 ± 0.27 | 0.392 |
| *IGF1* | 9.06± 0.63 | **0.0193** |  | 0.64 ± 1.07 | 0.383 |  | 0.09 ± 0.45 | 0.891 |
| *IGF1R* | 4.00± 0.39 | 0.5823 |  | -0.51 ± 0.21 | **0.0814** |  | -0.33 ± 0.45 | 0.461 |
| *IGFBP3* | 1.39± 0.86 | **0.0025** |  | 1.08 ± 0.64 | **0.0379** |  | 0.18 ± 0.52 | 0.724 |
| *IGFBP5* | 3.45± 0.32 | **0.02** |  | 0.14 ± 0.51 | 0.5714 |  | 0.04 ± 0.34 | 0.889 |
| *INSIG1* | 3.47± 0.33 | 0.3462 |  | -0.54 ± 0.25 | **0.0788** |  | 0.15 ± 0.31 | 0.707 |
| *LCA5L* | 4.26± 0.73 | **0.0005** |  | 1.90 ± 1.32 | **0.0002** |  | -0.34 ± 0.28 | 0.389 |
| *MAOB* | -2.81± 0.68 | **0.0011** |  | 1.45 ± 1.04 | **0.0013** |  | 0.23 ± 0.39 | 0.637 |
| *MAPK10* | -2.41± 0.51 | **0.0003** |  | 1.74 ± 1.2 | **0.0008** |  | -0.50 ± 0.29 | 0.237 |
| *MAPK14* | 0.63± 0.26 | **0.0931** |  | -0.22 ± 0.29 | 0.4946 |  | 0.42 ± 0.47 | 0.413 |
| *MAPK3* | -1.59± 0.49 | **0.0009** |  | 0.9 ± 1.52 | **0.0089** |  | 0.49 ± 1.21 | 0.312 |
| *MMP1* | -1.14± 0.43 | **0.0212** |  | 1.45 ± 0.21 | **<.0001** |  | -0.11 ± 0.86 | 0.730 |
| *MMP14* | -0.84± 0.29 | **0.0807** |  | -0.75 ± 0.42 | **0.0067** |  | -0.57 ± 0.35 | 0.146 |
| *MMP2* | -1.32± 0.51 | **0.0821** |  | 1.02 ± 1.25 | **0.0331** |  | 0.96 ± 0.75 | 0.111 |
| *NFKB1* | -1.39± 0.52 | **0.0646** |  | 0.51 ± 0.68 | 0.3052 |  | -0.33 ± 0.50 | 0.505 |
| *OCLN* | -0.38± 0.20 | 0.2685 |  | -0.43 ± 0.23 | **0.0453** |  | -0.80 ± 0.38 | **0.054** |
| *PCNA* | 0.96± 0.86 | 0.1505 |  | -1.93 ± 0.78 | **<.0001** |  | -0.16 ± 0.33 | 0.737 |
| *PIK3CB* | -1.11± 0.41 | **0.1156** |  | - | - |  | 0.47 ± 0.39 | 0.397 |
| *PIK3R2* | 0.69± 0.63 | **0.1077** |  | -0.63 ± 0.16 | **0.0108** |  | 1.54 ± 0.34 | **0.004** |
| *PRKACA* | 0.46± 0.35 | 0.3062 |  | -0.32 ± 0.7 | 0.3972 |  | 0.96 ± 0.51 | *0.059* |
| *PTGES* | -1.42± 0.42 | **0.0008** |  | 0.58 ± 0.86 | **0.0305** |  | -1.76 ± 0.51 | **0.017** |
| *RAC1* | 0.87± 0.70 | 0.2486 |  | -0.68 ± 0.82 | **0.0021** |  | 0.01 ± 0.42 | 0.981 |
| *RHEB* | -0.54± 0.29 | 0.1912 |  | -0.49 ± 1.18 | 0.1605 |  | 0.47 ± 0.44 | 0.215 |
| *RHOA* | 2.52± 1.45 | **0.0003** |  | -0.17 ± 1.4 | 0.6064 |  | -0.35 ± 0.41 | 0.375 |
| *SLC6A4* | -1.92± 0.37 | **0.0009** |  | 1.05 ± 1.53 | **0.0023** |  | -0.05 ± 0.31 | 0.893 |
| *SOCS3* | -2.56± 0.7 | **0.0009** |  | 2.92 ± 0.46 | **<.0001** |  | -0.44 ± 1.36 | 0.337 |
| *STAT3* | 0.02± 0.18 | 0.9286 |  | 0.05 ± 0.81 | 0.8688 |  | 0.42 ± 0.43 | 0.083 |
| *STAT5A* | -0.16± 0.49 | 0.663 |  | 1.01 ± 0.71 | **0.0073** |  | -0.01 ± 0.71 | 0.978 |
| *TEKT3* | -2.04± 0.46 | **0.0055** |  | 1.6 ± 1.44 | **0.0012** |  | -0.53 ± 0.33 | 0.266 |
| *TFEB* | -2.01± 0.63 | **0.0002** |  | 1.18 ± 1.26 | **0.0002** |  | -0.12 ± 0.29 | 0.723 |
| *TGFB1* | -1.31± 0.41 | **0.0509** |  | -0.34 ± 0.38 | 0.5184 |  | 0.45 ± 0.84 | 0.624 |
| *TJP1* | 0.43± 0.37 | 0.4313 |  | 0.12 ± 0.26 | 0.6762 |  | 0.51 ± 0.66 | 0.262 |
| *TJP2* | -2.12± 0.43 | **0.0002** |  | 1.32 ± 1.42 | **0.0001** |  | -0.20 ± 0.25 | 0.565 |
| *TJP3* | -0.51± 0.31 | 0.2493 |  | 1.52 ± 0.28 | **0.0004** |  | 0.53 ± 0.92 | 0.307 |
| *TNF* | -0.75± 0.78 | **0.0265** |  | 0.40 ± 0.35 | 0.4064 |  | -1.06 ± 0.5 | **0.031** |
| *TNFRSF1A* | -0.79± 0.37 | **0.0396** |  | 0.33 ± 0.57 | 0.3337 |  | -0.65 ± 0.83 | 0.120 |
| *TPH1* | -1.36± 0.78 | **0.0237** |  | 2.67 ± 0.31 | **<.0001** |  | 0.51 ± 1.16 | 0.219 |

**Supplementary Table 3.** Gene expression of primary bovine mammary epithelial cells (pBMEC) after 8-, 12- or 24-hour exposure to either 0, 50, 200 or 500 μM of 5-HTP. Data is presented as relative mRNA expression (∆∆Ct = ∆Ct Dose 0 (CON) - ∆Ct Dose 50, 200 or 500)) ± standard error of the mean (SEM). Positive and negative ∆∆Ct indicates upregulation and downregulation, respectively. Significance declared at *P*-value ≤ 0.05 (bold) and tendencies at 0.05 < *P*-value ≤ 0.10 (italicized).

|  | **0 *vs.* 50** | |  | **0 *vs.* 200** | |  | **0 *vs.* 500** | |
| --- | --- | --- | --- | --- | --- | --- | --- | --- |
| **Gene** | (∆∆Ct ± S.E) | P-value |  | (∆∆Ct ± S.E) | P-value |  | (∆∆Ct ± S.E) | P-value |
| **8 Hour** |  |  |  |  |  |  |  |  |
| **Apoptosis** |  |  |  |  |  |  |  |  |
| *BCL2* | -1.7 ± 0.81 | *0.07* |  | -1.81 ± 0.81 | **0.04** |  | -1.9 ± 0.81 | **0.05** |
| **Cell Proliferation** | |  |  |  |  |  |  |  |
| *MAPK10* | -2.11 ± 0.68 | **0.04** |  | -1.98 ± 0.68 | **0.05** |  | -1.41 ± 0.68 | 0.27 |
| **12 Hour** |  |  |  |  |  |  |  |  |
| **Apoptosis** |  |  |  |  |  |  |  |  |
| *IGFBP3* | 0.34 ± 0.76 | 0.65 |  | -1.19 ± 0.76 | **0.1** |  | -0.86 ± 0.76 | 0.24 |
| *TGFB1* | -1.1 ± 0.59 | 0.15 |  | -1.4 ± 0.59 | **0.08** |  | -1.85 ± 0.59 | **0.02** |
| **Cell Proliferation** | |  |  |  |  |  |  |  |
| *IGF1R* | 1.2 ± 0.29 | **0.004** |  | 1.3 ± 0.29 | **0.003** |  | 1.25 ± 0.29 | **0.006** |
| *CDKN1B* | - 0.02 ± 0.57 | 0.98 |  | -1.52 ± 0.57 | **0.05** |  | -0.98 ± 0.57 | 0.2 |
| *AKT2* | 0.14 ± 0.54 | 0.72 |  | 1.12 ± 0.55 | **0.01** |  | 0.72 ± 0.53 | *0.08* |
| *PRKACA* | 1.01 ± 0.76 | *0.07* |  | 0.40 ± 0.76 | 0.45 |  | 1.2 ± 0.76 | **0.03** |
| **Heat Shock** |  |  |  |  |  |  |  |  |
| *HSF1* | 0.09 ± 0.8 | 0.83 |  | -0.2 ± 0.8 | 0.64 |  | -0.82 ± 0.8 | *0.06* |
| **24 Hour** |  |  |  |  |  |  |  |  |
| **Serotonin Signaling** | |  |  |  |  |  |  |  |
| *5-HT7* | -0.58 ± 0.59 | 0.22 |  | -1.18 ± 0.59 | **0.01** |  | -0.37 ±0.59 | 0.44 |
| *AADC* | -2.46 ± 0.84 | **0.007** |  | -1.29 ± 0.83 | 0.13 |  | 0.09 ± 0.85 | 0.91 |
| *ALDH2* | 0.38 ± 0.5 | 0.55 |  | -0.98 ± 0.5 | *0.1* |  | -0.73 ± 0.5 | 0.25 |
| **Apoptosis** |  |  |  |  |  |  |  |  |
| *BAX* | -0.79 ± 0.43 | 0.17 |  | -1.68 ± 0.43 | **0.004** |  | -0.81 ± 0.43 | 0.17 |
| *BCL2* | - 0.7 ± 0.54 | 0.23 |  | -1.26 ± 0.54 | **0.03** |  | -0.33 ± 0.54 | 0.57 |
| *AIFM1* | 0.07 ± 0.39 | 0.9 |  | -1.41 ± 0.39 | **0.01** |  | -0.44 ± 0.41 | 0.44 |
| **Autophagy** |  |  |  |  |  |  |  |  |
| *ATG5* | 0.29 ± 0.45 | 0.61 |  | -1.24 ± 0.46 | **0.04** |  | 0.03 ± 0.48 | 0.96 |
| **Cell Proliferation** | |  |  |  |  |  |  |  |
| *CDKN1B* | -1.07 ± 0.52 | *0.1* |  | -0.89 ± 0.53 | 0.19 |  | -1.57 ± 0.56 | **0.02** |
| *CCND1* | 0.41 ± 0.49 | 0.47 |  | -0.70 ± 0.49 | 0.21 |  | -1.03 ± 0.51 | *0.09* |
| *PCNA* | 0.4 ± 0.48 | 0.95 |  | -1.6 ± 0.49 | **0.02** |  | -1.69 ± 0.48 | **0.01** |
| *AKT2* | -0.64 ± 0.71 | 0.33 |  | -1.57 ± 0.71 | **0.01** |  | -1.03 ± 0.72 | 0.13 |
| *PIK3R2* | 0.78 ± 0.55 | 0.29 |  | -1.05 ± 0.55 | *0.1* |  | -0.14 ± 0.55 | 0.85 |
| *RHEB* | - 0.33 ± 0.52 | 0.54 |  | -1.28 ± 0.52 | **0.01** |  | -0.35 ± 0.52 | 0.52 |
| *MAPK10* | -1.11 ± 0.44 | *0.07* |  | -1.54 ± 0.44 | **0.01** |  | -0.82 ± 0.44 | 0.18 |
| *RHOA* | 0.27 ± 0.51 | 0.63 |  | -0.92 ± 0.51 | *0.1* |  | -1.1 ± 0.51 | *0.06* |
| **Heat Shock** |  |  |  |  |  |  |  |  |
| *HSF1* | -0.31 ± 0.42 | 0.59 |  | -1.4 ± 0.42 | **0.01** |  | -0.96 ± 0.42 | *0.1* |
| **ECM Remodeling** | |  |  |  |  |  |  |  |
| *MMP14* | 0.6 ± 0.59 | 0.67 |  | -1.03 ± 0.59 | **0.004** |  | 0.23 ± 0.61 | 0.25 |
| *MMP2* | 1.75 ± 0.97 | *0.07* |  | 0.12 ± 0.97 | 0.89 |  | 0.77 ± 0.97 | 0.39 |
| **Tight Junctions** | |  |  |  |  |  |  |  |
| *TJP1* | 0.005 ± 0.74 | 0.99 |  | -1.009 ± 0.74 | 0.13 |  | -1.22 ± 0.74 | *0.06* |

**Supplemental Table 4.** Interaction between 5-Hydroxy-L-tryptophan (5-HTP) dose and incubation temperature. Gene expression of primary bovine mammary epithelial cells (pBMEC) after 8-, 12- or 24-hour exposure of either 0, 50, 200 or 500 μM doses of 5-HTP in thermoneutral (37°C, TN) or heat shock (41.5°C, HS) culture conditions. Data is presented as ∆∆Ct (∆∆Ct = ∆Ct TN Dose 0 (CON) - ∆Ct treatment)) ± standard error of the mean (SEM) with fold change (2^-∆∆Ct^) provided if trending towards significance or significant. Significance declared at *P*-value ≤ 0.05 and tendencies at 0.05 < *P*-value ≤ 0.10 (italicized).

|  | **TN** | | | | | | | |  | **HS** | | | | | | | |
| --- | --- | --- | --- | --- | --- | --- | --- | --- | --- | --- | --- | --- | --- | --- | --- | --- | --- |
|  | **0 *vs.* 50** | |  | **0 *v.s.* 200** | |  | **0 *v.s.* 500** | |  | **0 *v.s.* 50** | |  | **0 *v.s.* 200** | |  | **0 *v.s.* 500** | |
|  | ∆∆Ct ± S.E | FC |  | ∆∆Ct ± S.E | FC |  | ∆∆Ct ± S.E | FC |  | ∆∆Ct ± S.E | FC |  | ∆∆Ct ± S.E | FC |  | ∆∆Ct ± S.E | FC |
| **8 Hour** |  |  |  |  |  |  |  |  |  |  |  |  |  |  |  |  |  |
| **Serotonin Signaling** |  |  |  |  |  |  |  |  |  |  |  |  |  |  |  |  |  |
| *5-HT2C* | -0.17 ± 1.6 | - |  | -1.52 ± 1.6 | - |  | 0.16 ± 1.7 | - |  | -6.26 ± 1.9 | -77 |  | -1.5 ± 1.2 | - |  | -2.22 ± 1.7 | - |
| **Cell Proliferation** |  |  |  |  |  |  |  |  |  |  |  |  |  |  |  |  |  |
| *PCNA* | 1.83 ± 1.2 | - |  | 3.32 ± 1.2 | 9.9 |  | 3.19 ± 1.6 | 9.1 |  | 2.47 ± 1.2 | 5.5 |  | 2.93 ± 1.2 | - |  | 2.76 ± 1.3 | 6.7 |
| **12 Hour** |  |  |  |  |  |  |  |  |  |  |  |  |  |  |  |  |  |
| **Serotonin Signaling** |  |  |  |  |  |  |  |  |  |  |  |  |  |  |  |  |  |
| *5-HTR1A* | 1.00 ± 0.6 | *2.0* |  | 0.11 ± 0.6 | - |  | -0.39 ± 0.6 | - |  | 1.04 ± 0.6 | 2.1 |  | 1.56 ± 0.6 | 2.9 |  | 1.93 ± 0.6 | 3.8 |
| *5-HTR1B* | 1.06 ± 0.6 | *2.8* |  | 0.32 ± 0.6 | - |  | 0.58 ± 0.6 | - |  | 1.18± 0.6 | 2.3 |  | 1.58 ± 0.6 | 2.9 |  | 1.78 ± 0.6 | 3.4 |
| *5-HTR1F* | 1.04 ± 0.6 | *2.0* |  | 0.10 ± 0.6 | - |  | -0.27 ± 0.6 | - |  | 1.19 ± 0.6 | 2.3 |  | 1.62 ± 0.6 | 3.1 |  | 1.92 ± 0.6 | 3.8 |
| *5-HTR2B* | 0.98 ± 0.6 | - |  | 0.003± 0.6 | - |  | -0.46 ± 0.6 | - |  | 0.83 ± 0.6 | - |  | 1.27 ± 0.6 | 2.4 |  | 1.68 ± 0.6 | 3.2 |
| *5-HTR3C* | 1.06 ± 0.6 | *2.1* |  | 0.22 ± 0.6 | - |  | -0.24 ± 0.6 | - |  | 1.28 ± 0.6 | 2.4 |  | 1.79 ± 0.6 | 3.5 |  | 2.02 ± 0.7 | 4.1 |
| *SLC6A4* | 0.99 ± 0.6 | - |  | 0.25 ± 0.6 | - |  | -0.56 ± 0.6 | - |  | 0.66 ± 0.6 | - |  | 1.42 ± 0.7 | 2.7 |  | 1.46 ± 0.6 | 2.8 |
| *ALDH2* | -0.45 ± 0.4 | - |  | -0.28 ± 0.4 | - |  | -0.08 ± 0.4 | - |  | -1.37 ± 0.4 | -2.5 |  | -1.37 ± 0.5 | -2.6 |  | -1.19 ± 0.4 | -2.3 |
| *IDO1* | 1.06 ± 0.6 | - |  | 0.08 ± 0.7 | - |  | -0.41 ± 0.7 | - |  | 1.15 ± 0.6 | 2.2 |  | 1.62 ± 0.7 | - |  | 1.91 ± 0.6 | 3.8 |
| **Involution Biomarkers** | |  |  |  |  |  |  |  |  |  |  |  |  |  |  |  |  |
| *FASLG* | 6.43 ± 1.7 | 86.2 |  | 1.91 ± 1.5 | - |  | 2.78 ± 1.2 | 6.8 |  | 2.61 ± 1.2 | 6.1 |  | 5.27 ± 1.4 | 38.6 |  | 3.98 ± 1.4 | 15.8 |
| *STAT5A* | 0.48 ± 0.6 | - |  | 1.01 ± 0.6 | - |  | 0.98 ± 0.7 | - |  | 2.36 ± 0.6 | 5.1 |  | 1.07 ± 0.7 | - |  | 0.39 ± 0.7 | - |
| **Autophagy** |  |  |  |  |  |  |  |  |  |  |  |  |  |  |  |  |  |
| *ATG5* | -0.25 ± 0.3 | - |  | -0.37 ± 0.3 | - |  | 0.03 ± 0.4 | - |  | -0.18 ± 0.3 | - |  | -1.41 ± 0.4 | -2.7 |  | 0.11 ± 0.4 | - |
| *MAPK14* | -0.33 ± 0.6 | - |  | -0.93 ± 0.6 | - |  | -0.05 ± 0.6 | - |  | 0.51 ± 0.6 | - |  | -0.12 ± 0.7 | - |  | -1.13 ± 0.7 | *-2.2* |
| **Cell Proliferation** |  |  |  |  |  |  |  |  |  |  |  |  |  |  |  |  |  |
| *AKT1* | 1.35 ± 1.0 | - |  | 0.17 ± 1.0 | - |  | -0.19 ± 1.1 | - |  | 1.25 ± 1.0 | - |  | 1.28 ± 1.0 | - |  | 2.93 ± 1.1 | 7.6 |
| *FOXO3* | 0.95 ± 0.6 | - |  | 0.20 ± 0.6 | - |  | 0.49 ± 0.6 | - |  | 0.99 ± 0.6 | - |  | 1.37 ± 0.6 | 2.6 |  | 1.73 ± 0.7 | 3.3 |
| **Metabolic Regulator** |  |  |  |  |  |  |  |  |  |  |  |  |  |  |  |  |  |
| *INSIG1* | 1.22 ± 0.6 | 2.3 |  | -0.31 ± 0.6 | - |  | -0.28 ± 0.6 | - |  | -1.17 ± 0.6 | *2.3* |  | -0.83 ± 0.6 | - |  | 0.31 ± 0.6 | - |
| **Tight Junctions** |  |  |  |  |  |  |  |  |  |  |  |  |  |  |  |  |  |
| *TJP2* | 1.01 ± 0.6 | - |  | -0.18 ± 0.6 | - |  | -0.36 ± 0.6 | - |  | 1.04 ± 0.6 | *2.1* |  | 1.48 ± 0.6 | 2.8 |  | 1.86 ± 0.7 | 3.6 |
| *OCLN* | -0.18 ± 0.3 | - |  | -0.18 ± 0.3 | - |  | 0.36 ± 0.3 | - |  | 0.31 ± 0.4 | - |  | -0.79 ± 0.4 | *-1.7* |  | -1.47 ± 0.4 | -2.8 |
| **24 Hour** |  |  |  |  |  |  |  |  |  |  |  |  |  |  |  |  |  |
| **Autophagy** |  |  |  |  |  |  |  |  |  |  |  |  |  |  |  |  |  |
| *ATG3* | 1.47 ± 0.8 | *2.8* |  | -1.29 ± 0.7 | - |  | 0.20 ± 0.8 | - |  | -0.47 ± 0.8 | - |  | -0.6 ± 0.8 | - |  | -0.1 ± 0.9 | - |
| **Cell Proliferation** |  |  |  |  |  |  |  |  |  |  |  |  |  |  |  |  |  |
| *IGF1R* | 1.21 ± 0.9 | - |  | -1.23 ± 0.8 | - |  | 1.77 ± 0.8 | 3.4 |  | 1.30 ± 0.9 | - |  | -0.8 ± 0.8 | - |  | 1.2 ± 1.0 | - |
|  |  |  |  |  |  |  |  |  |  |  |  |  |  |  |  |  |  |
